# The moderating role of ethical awareness in the relationship between nurses’ artificial intelligence perceptions, attitudes, and innovative work behavior: a cross-sectional study

**DOI:** 10.1186/s12912-024-02143-0

**Published:** 2024-07-18

**Authors:** Amal Diab Ghanem Atalla, Ayman Mohamed El-Ashry, Samia Mohamed Sobhi Mohamed

**Affiliations:** 1 Nursing Administration, Faculty of Nursing, Alexandria University, Alexandria, Egypt; 2 Psychiatric and Mental Health Nursing, Faculty of Nursing, Alexandria University, Alexandria, Egypt; 3 Nursing Administration, Faculty of Nursing, Alexandria University, Alexandria, Egypt

**Keywords:** Artificial intelligence, Ethical awareness, Innovative work behavior, Nurses

## Abstract

**Background:**

Artificial intelligence is rapidly advancing and being integrated into healthcare, potentially revolutionizing patient care and improving outcomes by leveraging large datasets and complex algorithms.

**Aim:**

Investigate the moderating role of ethical awareness between nurses’ artificial intelligence perceptions, attitudes, and innovative work behaviors.

**Design and Methods:**

A cross-sectional descriptive correlational design adhering to STROBE guidelines. A non-probability convenience sample of 415 Alexandria Main University Hospital nurses was analyzed. Statistical methods included one-way ANOVA, the student t-test, and the Pearson coefficient, with results evaluated for significance at the 5% level and internal consistency assessed via Cronbach’s α. Linear regression assessed the predicting and moderating effect between ethical awareness, nurses’ artificial intelligence perceptions, attitudes, and innovative work behavior. The perceptions of using the Artificial Intelligence Scale, general attitudes towards the Artificial Intelligence Scale, ethical awareness of Using Artificial Intelligence, and the Employee Innovative Behavior Scale were used to respond to the research aim.

**Results:**

The study revealed that perception of AI use among nurses has a mean score of 50.25 (SD = 3.49), attitudes towards AI have a mean score of 71.40 (SD = 4.98), ethical awareness regarding AI use shows a mean score of 43.85 (SD = 3.39), and nurses innovative behavior exhibits a mean score of 83.63 (SD = 5.22). Attitude and ethical awareness were statistically significant predictors of innovation. Specifically, for every one-unit increase in attitude, innovative work behaviors increase by 1.796 units (*p* = 0.001), and for every one-unit increase in ethical awareness, innovative work behaviors increase by 2.567 units (*p* = 0.013). The interaction effects between perception, ethical awareness, attitude, and ethical awareness were also examined. Only the interaction between attitude and ethical awareness was found to be significant (*p* = 0.002), suggesting that the effect of attitude on innovative work behaviors depends on the level of ethical awareness. In other words, ethical awareness moderates the relationship between attitudes and innovative work behaviors rather than perception and innovation.

**Conclusion:**

There is a statistically significant correlation between attitude, ethical awareness, and creativity, highlighting that ethical awareness moderates the relationship between attitudes and innovative work behaviors. These findings emphasize the importance of ethical awareness in fostering positive attitudes towards AI and enhancing innovative practices in nursing, ultimately contributing to nurses’ well-being.

## Introduction

Artificial intelligence (AI) technologies are becoming increasingly prevalent in several organizations and sectors of society, including healthcare [1]. These technologies can completely transform administrative processes in healthcare, insurance, pharmaceutical organizations, and many aspects of patient care. AI is utilized increasingly in healthcare settings due to the complexity and abundance of data in these processes [2].

The potential benefits of AI applications in healthcare are immense. AI has the power to not just react to health issues, but to anticipate and prevent them. From identifying illnesses and customizing care, to real-time health issue tracking and anticipating patient needs, AI can enable a proactive approach to healthcare. It can also oversee the administrative side of healthcare delivery, potentially improving patient outcomes, enhancing accessibility, and supporting long-term healthcare systems [3–5].

Several key challenges must be addressed to ensure AI’s success in health organizations. Firstly, there needs to be more knowledge about the capabilities of various AI technologies among healthcare professionals and organizations; Sommer, Schmidbauer, and Wahi (2024), who’s revealed that knowledge about AI is limited, as only 25.2% can be described as AI experts [6].

A survey of 487 pathologists from 54 countries was employed by Sarwar et al. (2019) to investigate views on the application of AI in clinical practice. The results showed that participants’ opinions and attitudes toward AI were largely positive, with almost 75% expressing enthusiasm or curiosity about the technology as a diagnostic tool to enhance the effectiveness and quality of pathology workflows. Approximately 80% of participants anticipated using AI technology in pathology labs in the upcoming years [7].

This knowledge gap makes it difficult to fully leverage AI’s potential. However, the urgency to address these challenges is clear. Many organizations need clearer strategies for integrating AI into their healthcare systems to tackle pressing issues effectively. Compounding these challenges is a need for more personnel trained to implement and manage AI technologies. Furthermore, AI technologies often need more compatibility with legacy infrastructure, hindering seamless integration. Finally, more access to high-quality and diverse data is needed to ensure the effective functioning of AI systems. Addressing these obstacles is not just important but essential for successfully adopting and implementing AI in healthcare [8, 9].

While there are many obstacles to overcome when applying AI in healthcare, ethical considerations are the most important. AI has brought up ethical issues that need to be acknowledged and properly handled in the most effective and scientifically supported manner feasible. AI in healthcare raises a wide range of complex ethical issues. While recent analyses of the implications of AI in public health have suggested that more research be done to ensure the ethical design and deployment of AI and that patients should be more cautious when introducing AI into healthcare, AI may have the potential to improve individual health as well as increase the resilience and sustainability of health systems [10].

In 2021, the WHO released a report on the application of AI systems in healthcare [11]. The WHO’s studies include important guidelines and points of concern for the ethical and responsible application of AI systems. It has been proven that AI for health should be developed and applied to uphold basic human rights, dignity, and moral principles. AI systems should not only encourage but also demand accountability, responsibility, safety, justice, equity, transparency, and inclusivity [12].

**Fig. 1:**
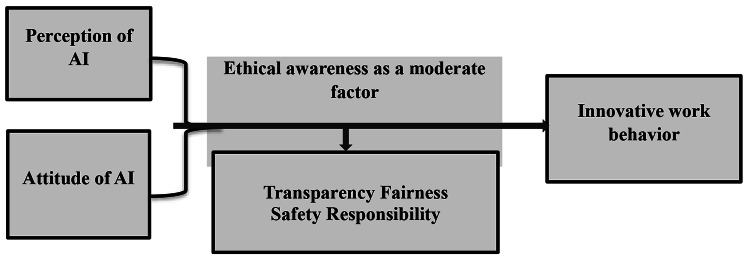
The conceptual framework proposed by the researchers: Ethical Awareness as a Moderate Nurses’ Artificial Intelligence Perceptions, Attitudes and Innovative Work Behavior

AI ethics awareness and attitude toward AI affect nursing behavior [13]. AI has been proposed to boost nurses’ creativity, which boosts productivity [14], as shown in a conceptual model Fig. 1. Healthcare providers that engage in innovative work behavior (IWB) generate and implement new, imaginative solutions to challenges to capitalize on opportunities and increase the competitiveness of the healthcare organization [15].

The application and implementation of innovative ideas are necessary for successful innovation. This is accomplished by modifying the concept or implementation strategies until the process or service is enhanced and applied within the healthcare organization [16]. Within the categories of idea creation, idea search, concept communication, execution beginning actions, including others, overcoming hurdles, and creative output, Lukes and Stephan (2017) categorized innovative work behavior. It’s crucial for nurses to not only adapt but also embrace the technological developments that have affected every aspect of their profession and changed daily schedules, customs, and working hours [16].

The emergence of AI and new developing technologies provide the framework for healthcare transformation. New labor standards and practices are additionally required. However, what sets this study apart is its focus on the attitudes and views of nurses toward AI, a novel and unexplored area. Healthcare organizations’ objectives also include providing high-quality care quickly, without regard to time or space constraints, reducing medical errors, using innovative methods of operation, and minimizing physical tiredness [17, 18].

The significance of this study is pertinent to Egypt’s nursing industry. It fills a significant vacuum in the literature by emphasizing the ethical significance of nurses’ knowledge of AI and how it affects creative work practices. For example, Egyptian research revealed that no management in the medical or nursing fields was familiar with artificial intelligence. Nurse Managers are typically in favor of most things when it comes to the usage of AI in nursing. According to the same trend, most patients favor employing AI in healthcare [16]. This emphasizes how crucial nurses’ attitudes and ethical awareness will be in determining future healthcare direction.

Additionally, Technology integration in healthcare can completely transform medical procedures by providing more precise diagnoses, individualized treatment plans, and better patient outcomes. However, the quick development of AI in healthcare also brings up important ethical issues that must be thoroughly considered and resolved [19]. The study offers insight into the complex interplay between nurses’ AI perceptions, attitudes, innovative work behavior, and the moderating influence of ethical awareness. These findings can enlighten the development of evidence-based policy strategies and support AI’s accountable and operative integration in nursing practice.

Concerning numerous studies, several ethical concerns related to the use of AI in healthcare need to be carefully considered. It is imperative to tackle concerns related to privacy, transparency, bias, patient autonomy, safety, and workforce effects to promote the conscientious and advantageous utilization of AI technology. In this endeavor, Collaboration is necessary to address these ethical issues and advance AI’s responsible and beneficial application in healthcare. To do this, healthcare professionals, legislators, ethicists, and technologists must collaborate while offering their distinct viewpoints and areas of expertise.

 [11, 18, 20, 21]. Furthermore, Zirar (2023) demonstrated that by fostering creativity and serving as a versatile tool for innovation, artificial intelligence (AI) has the potential to enhance workers’ productivity [22]. Hence, for nurses to enhance their knowledge and deliver high-quality care, it is crucial for them to understand AI and ethical concepts and to have a positive attitude towards them. This will empower them to play a pivotal role in this collaborative endeavor, thereby underscoring the significance of their involvement in the integration of AI in healthcare.

### Aim of the study

Investigate the moderating role of ethical awareness between artificial intelligence perceptions, attitudes, and innovative work behavior?

### Research hypothesis

Ethical awareness is moderates the relationship between artificial intelligence perceptions, and attitudes, innovative work behavior?

### Study design

As per STROBE principles, a cross-sectional descriptive design was used.

### Setting

The investigation used a descriptive correlational method. The current study was conducted throughout all medical, surgical, and critical care inpatient wards at Alexandria Main University Hospital in Egypt. This hospital provides free public health services. It is the largest university hospital in the governorate of Alexandria. With 6,658 beds and a wide range of nursing specialties, including professional, technical, and diploma nurses, the hospital is regarded as the largest provider of healthcare services.

Additionally, patients from all governorates in Egypt can receive a comprehensive range of healthcare services from this facility, including inpatient, outpatient, critical, intense, emergency care, radiographic, laboratory, and physical therapy services. There are 775 beds in surgical care units with their respective specialties, 953 beds in medical care, and 101 beds in critical care units. There are 17 surgical care units with various specialties, 25 medical care units with various specialties, and 13 critical care units.

### Sample size and sampling

The G*Power Windows 3.1.9.7 software was used to estimate the sample size for the study. The calculation was based on the following parameters: a power (1-β error probability) of 0.95, an effect size of 0.5, and an α-error probability of 0.01, with one group and three predictors. According to the software, a sample size of 415 nurses was required for the study [23]. Subsequently, a non-probability convenience sample of 415 nurses was used in the study. This practical and accessible sampling technique allowed for the inclusion of nurses who were readily available and willing to participate and had worked in the designated units for at least six months. The sample was divided into three groups based on the type of unit they worked in 150 nurses from medical care units, 120 from surgical care units, and 145 from critical care units. Despite not being randomly selected, this sampling technique was chosen for its practicality and ease of access, which may limit the generalizability of the results.

### Study measurements

#### Tool I: Socio-demographic characteristics

The study participants’ years of service, years in the work unit, gender, age, education, and nursing experience were among the topics the researchers questioned.

#### Tool II: perception of the use of artificial intelligence scale

It was created by Oh et al. (2019) [24] to find out how doctors felt about applying AI applications. Abdullah & Fakieh (2020) [25] revised the questionnaire, removing questions about pure medication and switching the multiple-choice questions to a Likert scale. This was done because the original questionnaire was intended for doctors. The final version of this study’s 14-item, three-section questionnaire was adapted from Abdullah & Fakieh (2020) [25]. There were four items in the first segment (perceptions of AI). Five items were in the second part (the benefits of utilizing AI). There were five items in the last part, “Problems for AI application in health care.” A 5-point Likert scale, ranging from strongly agree (5) to strongly disagree (1), was used to measure the replies. The total score falls between 14 and 70. The present study’s Cronbach alpha was 0.92.

#### Tool III: the general attitudes towards artificial intelligence scale (GAAIS)

The researcher modified an instrument developed by Schepman & Rodway (2020) [26]. It has two subscales (20 items total): eight negative and twelve positive items on AI. Currently, the factor structure has strong construct validity because the positivity and negativity of the assumed items throughout their formation were statistically supported. A 5-point Likert scale, ranging from strongly agree (5) to strongly disagree (1), was used to measure the replies. The total score falls between 20 and 100. Cronbach’s alpha for the current investigation was 0.810.

#### Tool IV: ethical awareness to use artificial intelligence

Ko & Leem (2021) [27] created this instrument to gauge nurses’ ethical perceptions of their intended usage of AI. There were twelve total items, divided into four dimensions (three items each): accountability, safety (de-hazard), transparency, and justice. A 5-point Likert scale, with 5 representing strongly agree and 1 representing strongly disagree, was used to measure the replies. The total score is between 12 and 60. Cronbach’s alpha for the current investigation was 0.88.

#### Tool V: employee innovative behavior scale

Lukes & Stephan (2017) [16] created this measure to evaluate innovative behavior among employees. It had the following seven dimensions and twenty-three items: Three components are related to idea generation, three to idea search, four to idea communication, and three to implementation beginning actions, three to involving others, four to overcoming hurdles, and three to innovative work behaviors outputs. The responses were measured using a 5-point Likert scale: five represents strongly agree, and one represents strongly disagree. The overall score ranges from 12 to 60. For the current study, Cronbach’s alpha was 0.90.

## Methods

### Ethical considerations

The Nursing Research Ethics Committee of Alexandria University’s Faculty of Nursing in Egypt approved the study procedure and guaranteed that the investigation complied with ethical guidelines, with reference number IRB00013620/AU/20-8-23, on 20th August 2023. The study’s goal was clearly disclosed to the nurses, and their agreement was acquired. Each questionnaire was given a code number to safeguard the respondents’ confidentiality and identity. As agreed upon with the nurses, the data was only utilized for the study. The opportunity to opt out was also confirmed to further guarantee the study’s ethical conduct.

### Tools validity

Two independent translators, fluent in English and Arabic, translated the tools from English to Arabic. The two translations were then compared and reconciled by a committee or a third translator to create a consensus version, ensuring the translation accurately captured the original meaning. Next, the consensus Arabic version was back-translated into English by two independent translators who had yet to see the original English version. The back-translated English versions were compared with the original English text to identify discrepancies or inconsistencies. Differences were discussed and resolved to ensure the Arabic version accurately reflected the original content. A panel of seven professor experts (Five from nursing administration and two from psychiatric nursing), including bilingual professionals and subject matter experts, reviewed the final Arabic version for cultural relevance, clarity, and content accuracy. This step ensured the translation was appropriate for the target audience and retained the intended meaning. The translated tools were then pre-tested with a small sample from the target population to identify any comprehension, language, or cultural relevance issues. Feedback from this pre-testing phase was used to make final adjustments to the translation. Finally, the translated and reviewed tools were finalized, incorporating all feedback to ensure they were ready for use in the study [28, 29].

### Pilot study

10% of nurses (*n* = 42) endorsed the pilot research to maintain the goods’ simplicity and practicality by identifying potential issues and roadblocks during data collection. Nothing needed to be altered. The study did not include those who took part in the pilot study. The researchers checked the questionnaires for accuracy and inclusivity.

### Data collection

Personal copies of the questionnaires were given to the research subjects. The researchers gave each nurse a hand-delivered questionnaire and then picked up the completed forms. Each nurse was given a ten-minute explanation of the study’s goal before being asked to return it to the researcher. These scales were completed 20–30 min before the researcher to confirm the respondents’ objectivity, the coherence of their thoughts, and the completion of all questions. Because they were connected to distinct working units, it was easy to monitor the distribution and collection to guarantee the highest response rate. Participants received little treats as a thank-you for their participation. Completing the questions should take fifteen to twenty minutes.

### Data quality management

The researchers encompass a range of activities to improve overall data quality throughout the research cycle. This involves identifying and addressing data anomalies, errors, redundancies, and inconsistencies and enhancing data accuracy and integrity. The process includes data profiling, cleansing, validation, and monitoring, among other key practices.

### Data analysis

The IBM SPSS software program version 23.0 was utilized for data analysis once the data was imported into the computer. Initially, normality tests, including the Shapiro-Wilk and Kolmogorov-Smirnov tests, were conducted to assess whether the data followed a normal distribution. Upon confirming that the data met the normality criteria, parametric methods were employed for further analysis. Descriptive statistics, including means, standard deviations, frequencies, and percentages, were calculated to provide an overview of the sample characteristics and key variables. For comparative analysis, a one-way ANOVA test was used to compare more than two groups to determine if there were statistically significant differences among them, and the Student’s t-test was employed to compare two quantitative data categories that were regularly distributed, determining if there was a significant difference between the means of the two groups. The Pearson coefficient was utilized to examine the relationships between normally distributed quantitative data, helping to understand the strength and direction of linear relationships between variables. To assess the internal consistency of the scales used in the study, Cronbach’s α coefficient was computed, with a value above 0.70 generally indicating acceptable reliability. Linear regression was conducted to evaluate the predictive and moderating effects between ethical awareness, nurses’ perceptions of artificial intelligence, attitudes, and innovative work behavior. This regression analysis included checking for multicollinearity, ensuring the independence of errors, and evaluating the model’s overall fit using R-squared values. The significance of the results was evaluated at the 5% level (*p* < 0.05), with both one-tailed and two-tailed tests considered based on the specific hypotheses. One-tailed tests were used when the direction of the relationship was hypothesized, while two-tailed tests were used when no specific direction was hypothesized.

## Results

Table 1 provides demographic information about the 415 nurses studied. Most nurses are female (83.6%), with a mean age of 44.04 years with SD of 7.03. Most nurses are married (77.8%), and the qualifications are distributed as follows: 38.6% professional, 50.4% technical, and 11.1% practical. The mean years of nursing experience is 7.92 years with SD 3.87, and the mean years of hospital experience is 7.24 years with SD 3.81. Only 27.5% of the nurses have attended AI workshops.

**Table 1:** Distribution of the studied nurses according to demographic data (*n* = 415)

Demographic characteristics	No	%
**Sex**		
Male	68	16.4
Female	347	83.6
**Age (years)**		
20–30	6	1.4
30–40	117	28.2
40–50	225	54.2
> 50	67	16.1
Mean ± SD	44.04 ± 7.03	
**Marital status**		
Single	58	14.0
Married	323	77.8
Divorced	8	1.9
Widowed	26	6.3
**Qualification**		
Professional	160	38.6
Technical	209	50.4
Practical	46	11.1
**Experience year of nursing**		
1–5	93	22.4
5–10	224	54.0
10–15	86	20.7
More than 15	12	2.9
Mean ± SD	7.92 ± 3.87	
**Experience hospital**		
1–5	90	21.7
5–10	227	54.7
10–15	86	20.7
More than 15	12	2.9
Mean ± SD	7.24 ± 3.81	
**Attendance AI workshops**		
Yes	114	27.5
No	301	72.5

Table 2 illustrates the distribution of the nurses based on their mean percent score in four key areas: perception, attitude, ethical awareness, and innovation. The perception of AI use among nurses indicates a generally average level of perception with a mean total score of 50.25 (SD = 3.49) and a mean percent score of 64.73% (SD = 6.23), suggesting that most nurses fall into the moderate category. The General Attitudes Towards Artificial Intelligence Scale (GAAIS) reveals that nurses’ attitudes towards AI are also moderate, with a mean total score of 71.40 (SD = 4.98) and a mean percent score of 64.25% (SD = 6.23), showing a balanced view with both positive and negative aspects considered. The ethical awareness regarding AI use was relatively high among the nurses, with a mean total score of 43.85 (SD = 3.39) and a mean percent score of 66.36% (SD = 7.07), indicating a strong awareness and consideration of ethical implications in AI usage. Employee Innovative Behavior shows that nurses exhibit a high level of innovative behaviors, with a mean total score of 83.63 (SD = 5.22) and a mean percent score of 65.90% (SD = 5.67), reflecting a moderately high engagement in innovation-related activities.

**Table 2:** Distribution of the studied nurses according to their levels and mean percent score (*n* = 415)

	Total score	Mean percent score
	Mean ± SD	Mean ± SD
**perception**	50.25 ± 3.49	64.73 ± 6.23
**Attitude**	**71.40 ± 4.98**	**64.25 ± 6.23**
**Ethical Awareness**	43.85 ± 3.39	66.36 ± 7.07
**Innovative work behaviors**	83.63 ± 5.22	65.90 ± 5.67

A thorough overview of the relationships between the variables under study is given in Table 3. Importantly, perception and attitude showed a substantial, moderate positive association (*r* = 0.315, *p* < 0.001), as did perception and ethical awareness (*r* = 0.276, *p* < 0.001), while attitude and ethical awareness showed a significant weak positive correlation (*r* = 0.163, *p* = 0.001). Additionally, there is a significant weak positive association (*r* = 0.239, *p* < 0.001), a strong, moderate positive correlation (*r* = 0.392, *p* < 0.001), and a substantial correlation (*r* = 0.191, *p* < 0.001) between innovative work behaviors and perception, attitude, and ethical awareness.

**Table 3:** Correlation between the studied variables (*n* = 415)

		Perception	Attitude	Ethical Awareness
**Attitude**	**r**	0.315*		
	**p**	< 0.001		
**Ethical Awareness**	**r**	0.276*	0.163*	
	**p**	< 0.001	0.001	
**Innovative work behaviors**	**r**	0.392*	0.239*	0.191*
	**p**	< 0.001	< 0.001	< 0.001

r: Pearson coefficient *: Statistically significant at *p* ≤ 0.05 Weak from 0.000 to 0.25 Moderate from > 0.25 to 0.75 Strong from > 0.75 to 1.00

Table 4 presents the relationship between demographic data and variables, including perception, attitude, ethical awareness, and innovation. Regarding sex, male nurses have higher mean scores in all variables than female nurses. The differences are statistically significant for perception (*p* = 0.003), ethical awareness (*p* = 0.046), and innovative work behaviors (*p* = 0.046). Nurses aged 30–40 years have the highest mean scores in perception, attitude, and innovation, while those over 50 have the lowest. The differences in perception across age groups are statistically significant (*p* < 0.001). Divorced nurses have the highest mean scores in all variables, while widowed nurses have the lowest. The differences in perception (*p* < 0.001) and ethical awareness (*p* = 0.006) across marital status are statistically significant. No statistically significant differences exist in perception, attitude, ethical awareness, and innovative work behaviors across different qualifications. Nurses with 1–5 years of experience have the highest mean scores in all variables, while those with more than 15 years of experience have the lowest. The differences in perception (*p* < 0.001), attitude (*p* = 0.049), and ethical awareness (*p* = 0.003) across different years of nursing experience are statistically significant. Nurses who attended any workshops in artificial intelligence AI had higher mean scores in all variables than those who did not. The differences are statistically significant for perception (*p* < 0.001), attitude (*p* = 0.035), and innovative work behaviors (*p* = 0.025).

**Table 4:** Relation between demographic data and different variables (*n* = 415)

Demographic characteristics	perception		Attitude		Ethical Awareness		Innovative work behaviors	
	Mean ± SD.	Test of sig	Mean ± SD.	Test of sig	Mean ± SD.	Test of sig	Mean ± SD.	Test of sig
**Sex**								
Male	51.24 ± 2.75	t = 3.067^*^	72.26 ± 4.34	t = 1.568	44.60 ± 2.92	t = 2.001^*^	84.71 ± 4.69	t = 2.022^*^
Female	50.05 ± 3.59	*p* = 0.003^*^	71.23 ± 5.09	*p* = 0.118	43.71 ± 3.46	*p* = 0.046^*^	83.42 ± 5.30	*p* = 0.046^*^
**Age (years)**								
20–30	52.00 ± 0.00		71.0 ± 4.38		44.67 ± 2.34		81.83 ± 6.59	
30–40	50.90 ± 3.63	F = 6.443^*^	71.35 ± 5.29	F = 1.042	43.68 ± 3.43	F = 0.660	84.07 ± 5.52	F = 1.175
40–50	50.32 ± 2.87	*p* < 0.001^*^	71.71 ± 4.44	*P* = 0.374	44.03 ± 3.11	*p* = 0.577	83.72 ± 4.91	*p* = 0.319
> 50	48.72 ± 4.66		70.49 ± 6.10		43.49 ± 4.23		82.75 ± 5.53	
**Marital status**								
Single	51.09 ± 2.70		71.48 ± 4.93		43.26 ± 3.02		83.38 ± 5.96	
Married	50.11 ± 3.37	F = 16.562^*^	71.16 ± 4.85	F = 2.522	43.94 ± 3.35	F = 4.244^*^	83.61 ± 4.99	F = 4.940^*^
Divorced	57.00 ± 0.00	*p* < 0.001^*^	75.13 ± 2.42	*P* = 0.057	47.38 ± 4.60	*p* = 0.006^*^	90.25 ± 2.25	*p* = 0.002^*^
Widowed	48.00 ± 4.14		69.77 ± 5.93		42.96 ± 3.74		82.42 ± 5.63	
**Qualification**								
Professional	50.64 ± 2.73	F = 1.695	71.64 ± 4.17	F = 0.311	43.70 ± 2.99	F = 0.365	84.01 ± 4.93	F = 0.777
Technical	49.97 ± 3.69	*p* = 0.185	71.24 ± 5.30	*P* = 0.733	43.90 ± 3.61	*p* = 0.695	83.45 ± 5.28	*p* = 0.461
Practical	50.17 ± 4.64		71.26 ± 6.07		44.15 ± 3.71		83.11 ± 5.91	
**Experience year of nursing**								
1–5	51.55 ± 3.52		71.74 ± 5.33		43.62 ± 3.41		83.44 ± 5.74	
5–10	50.17 ± 2.94	F = 19.812^*^	71.65 ± 4.50	F = 2.637*	44.06 ± 3.16	F = 4.741^*^	83.78 ± 5.03	F = 2.696^*^
10–15	49.93 ± 3.88	*p* < 0.001^*^	70.87 ± 4.89	*P* = 0.049*	44.03 ± 3.83	*p* = 0.003^*^	84.01 ± 5.02	*p* = 0.046^*^
More than 15	44.00 ± 2.09		67.92 ± 9.17		40.42 ± 2.35		79.58 ± 4.64	
**Attendance workshop about AI**								
Yes	51.53 ± 2.80	t = 5.271^*^	72.11 ± 3.61	t = 2.116*	44.06 ± 3.10	t = 0.770	84.56 ± 4.91	t = 2.246
No	49.76 ± 3.60	*P* < 0.001^*^	71.13 ± 5.39	*p* = 0.035*	43.77 ± 3.50	*p* = 0.442	83.28 ± 5.29	*p* = 0.025^*^

F: One way ANOVA test t: Student t-test *: Statistically significant at *p* ≤ 0.05

Table 5 comprehensively analyzes how perception, attitude, ethical awareness, and other covariates influence innovative work behaviors among 415 participants. The model, which rigorously accounts for 20.5% of the variance in innovative work behaviors (R²=0.205) and is statistically significant (F = 10.404, *p* < 0.001), is a testament to the robustness of our statistical analysis. Including interaction terms, which add a significant 2% to the explained variance (R² change = 0.020, F = 4.973, *p* = 0.007), further strengthens the validity of our findings. While some covariates such as sex, marital status, years of nursing experience, and AI workshop attendance were found to be non-significant, indicating they do not significantly influence innovative work behaviors, attitudes, and ethical awareness emerged as significant predictors. A positive attitude (B = 1.796, *p* = 0.001) is strongly associated with higher innovative work behaviors, and ethical awareness (B = 2.567, *p* = 0.013) significantly drives innovative behaviors. The significant interaction between attitude and ethical awareness (B = 0.038, *p* = 0.002) indicates that the relationship between attitude and innovative work behaviors is moderated by ethical awareness. Conversely, the interaction between perception and ethical awareness was insignificant (B = 0.005, *p* = 0.767), indicating no moderating effect on the relationship between perception and innovative work behaviors.

**Table 5:** Linear regression on the effect of perception, attitude, ethical awareness, and other covariates on innovative work behaviors (*n* = 415)

Predictors	B	SE	t	*p*	95% CI	
					LL	UL
**Constant**	-63.612	45.110	-1.410	0.159	-152.293	25.068
**Covariates**						
Sex (female)	-0.634	0.668	-0.949	0.343	-1.947	0.679
Marital status (Married)	0.058	0.306	0.190	0.849	-0.543	0.659
Experience year of nursing	-1.044	2.815	-0.371	0.711	-6.579	4.490
Attendance AI workshops	-0.139	0.547	-0.253	0.800	-1.214	0.937
**Main effect**						
Perception	0.285	0.804	0.355	0.723	-1.295	1.866
Attitude	1.796	0.550	3.265*	0.001*	0.715	2.877
Ethical Awareness	2.567	1.031	2.488*	0.013*	0.539	4.594
**Interaction effect**						
Perception x Ethical Awareness	0.005	0.018	0.297	0.767	-0.030	0.041
Attitude x Ethical Awareness	0.038	0.013	3.049*	0.002*	0.014	0.063

R^2^ = 0.205, F = 10.404*,*p* < 0.001*

R^2^ change = 0.020, F = 4.973*, *p* = 0.007*

R^2^: Coefficient of determination, B: Unstandardized Coefficients, SE: standard error, t: t-test of significance,

LL: Lower limit, UL: Upper Limit, CI: confidence interval, *: Statistically significant at *p* ≤ 0.05

## Discussion

The results of this study reveal insightful trends in nurses’ perceptions, attitudes, ethical awareness, and innovative behaviors regarding AI usage. Nurses exhibit an average mean of perception and attitude towards AI, reflecting a balanced view of its potential benefits and drawbacks. Ethical awareness among nurses is relatively high, with a mean percent score of 66.36%, indicating a strong consideration of ethical implications in AI use. Innovative work behaviors are also notably high, suggesting that nurses actively engage in innovative activities. The analysis highlights the moderating effect of ethical awareness on the relationship between attitude and innovative work behaviors. Conversely, the interaction between perception and ethical awareness was not significant. Thus, ethical awareness plays a crucial role in moderating the effects of positive attitudes towards innovation but does not similarly impact the relationship between perception and innovative behaviors.

The results indicate that nurses have a generally average perception and attitude toward artificial intelligence (AI). Ethical awareness, however, was relatively high. These findings suggest that while nurses are open to using AI in healthcare, they maintain a balanced view, recognizing potential benefits and ethical implications. Moreover, innovative work behaviors among nurses were moderately high, with a mean percent score of 65.90%. This indicates that nurses actively engage in innovation-related activities, which are crucial for improving healthcare practices and patient outcomes.

This study supports the findings of Serbaya et al. (2024) that healthcare professionals generally had positive attitudes and a good awareness of AI [30]. Furthermore, another research study revealed that most healthcare professionals had a good attitude about using AI in healthcare but did not consider it dangerous to their jobs. Most survey participants believed artificial intelligence is crucial to healthcare [31]. Additionally, this study is contracted with Elderiny et al. (2024), which shows that around two-thirds (66.2%) of studied nurses need a satisfactory level of knowledge of AI applications [32].

Moreover, there is a noteworthy moderately positive correlation between perception and innovation. However, regarding the linear regression model, perception of AI has little effect on innovation, but Attitude regarding AI and ethical Awareness were statistically significant predictors of innovation. This indicates that the perception of AI is only enough to gain innovative work behavior if gaining a positive attitude and adhering to ethical principles. Additionally, this could be explained by the fact that the implementation of AI in the work aspects can be used to find innovative ways to accomplish tasks at work, find and implement the best new ideas, and create appropriate plans and schedules for implementing new ideas.

According to Akinrinmade et al. (2023), this study shows that the application of AI has the potential to have a big impact on healthcare in the twenty-first century, promote progressive medical innovation, and foster creative growth [33]. Furthermore, enhanced knowledge of AI-based innovative work behavior management as it exists today, how it will influence innovative work behavior practices in the future, and how different organizations have opted to incorporate AI and their expectations for it [34].

In addition, this study shows a statistically significant difference between Perception of AI, ethical awareness, innovation, and gender, in which male nurses have higher mean scores in all variables than female nurses, which means male nurses are more aware of AI uses and applications used in healthcare organizations than female nurses. This finding aligns with the findings of Serbaya et al. (2024), which indicate that male healthcare professionals scored higher on AI knowledge tests than female healthcare workers (Beta = 0.555, 95%, p-value = 0.010) [30].

Additionally, nurses aged 30–40 years have the highest mean scores in perception, attitude, and innovation, while those over 50 have the lowest, indicating that elderly nurses prefer to adhere to traditional methods to perform tasks more than youth nurses. Moreover, nurses with 1–5 years of experience have the highest mean scores in all variables, while those with more than 15 years of experience have the lowest, indicating that newly graduated nurses are more aware of AI uses and ethical issues. The results of this study agree with Al-Sabawy (2023) that the age group of 26–30 years and those having 0–10 years of experience are the most likely to have a positive perception and attitude toward AI [35].

Moreover, no statistically significant differences in perception, attitude, ethical awareness, and innovative work behaviors across different qualifications. This study is contradicted by Al-Sabawy (2023), who revealed statistically significant differences in perception of AI and qualification level and found over half of the group holds a Bachelor of Science in Nursing (BScN) degree and have good AI perception, which indicates advanced academic qualifications may provide a broader or deeper exposure to technological advancements and their implications, fostering a more receptive stance [35].

Finally, the linear regression model suggests that the effect of attitude on innovative work behaviors depends on the level of ethical awareness. In other words, ethical awareness of transparency, fairness, safety, and responsibility moderates the relationship between attitudes and innovative work behaviors rather than perception and innovation. Along the same lines, according to Chang et al. (2023), adopting a positive attitude toward AI requires adherence to four ethical principles: transparency, fairness, privacy, and non-maleficence. According to the respondents, while implementing AI in the workplace, non-maleficence should take precedence over transparency, privacy, and justice [36].

### Limitations

The study faced several limitations that could impact the interpretation and generalizability of the findings. Firstly, using a non-probability convenience sample may limit the generalizability of the results as the sample was not randomly selected, potentially introducing selection bias and affecting representativeness. Additionally, the reliance on self-reported data may be subject to response biases such as social desirability bias, where participants might provide responses they believe are favorable rather than their true feelings or behaviors. Conducting the study in a single hospital further limits external validity, making it difficult to generalize the results to nurses in different hospitals or healthcare settings. Moreover, the study’s cross-sectional nature precludes the ability to track changes in attitudes and behaviors over time or assess the long-term impact of AI on innovative work behaviors. Without examining specific types or applications of AI, the general perceptions and attitudes toward AI may have included important nuances in nurses’ views and concerns. Finally, despite controlling for various covariates, other confounding variables may not be accounted for, which could influence the relationship between the studied factors and innovative work behaviors.

## Conclusion

By adhering to ethical standards that can be utilized to identify and execute the best new ideas, develop creative methods to complete duties at work, and build suitable plans and schedules for implementing new ideas, the findings highlight the necessity of applying AI in the work aspects. This study found a statistically significant correlation between attitude, ethical awareness, and creativity. We also examined how perception, ethical awareness, attitude, and awareness interacted. Only the interaction between attitude and ethical awareness was found to be significant, suggesting that the effect of attitude on innovative work behaviors depends on ethical awareness. In other words, ethical awareness moderates the relationship between attitudes and innovative work behaviors rather than perception.

### Implications in nursing practice

Initially, nurses’ inventive behavior is greatly enhanced by artificial intelligence. Second, having a positive AI mindset is necessary for nurses to uphold ethical standards. This research has consequences for healthcare organizations and legislators, who must prioritize the development and upkeep of AI, ethical consciousness, and creative work practices among nurses.

The study also emphasizes the importance of implementing comprehensive teaching initiatives and other interventions to enhance nurses’ perceptions of AI and attitudes. Healthcare organizations may create an atmosphere that supports the development and well-being of nurses by providing them with the tools, resources, and expertise they need. This can improve follow-up treatment for patients, reduce error rates, and increase work satisfaction for nurses. Improve innovative work behaviours and communication in healthcare settings by incorporating the ideas and opinions of nurses.

## Data Availability

The corresponding author can provide the datasets created and analyzed for this work upon reasonable request.

## Abbreviations

GAAIS: General Attitudes towards Artificial Intelligence Scale
AI: Artificial intelligence
IWB: Innovative Work Behavior
SD: Standard Deviation
